# Characterization of hawthorn pectin gained via different ethanol concentrations

**DOI:** 10.1002/fsn3.3321

**Published:** 2023-03-17

**Authors:** Haoyu Wang, Yiwei Zhu, Dan Li, Chuanhe Zhu

**Affiliations:** ^1^ Key Laboratory of Food Processing Technology and Quality Control in Shandong Province, College of Food Science and Engineering Shandong Agricultural University Tai'an China; ^2^ Shandong Medicine Technician College Tai'an China

**Keywords:** characterization, ethanol precipitation, hawthorn, pectin

## Abstract

Pectin is identified as an effective delivery material due to its excellent gel‐forming ability, low immunogenic properties, biocompatibility, and biodegradability. These excellent properties depend on the preparation method of pectin. In the study, four pectin fractions (named: CAHP30, CAHP40, CAHP50, and CAHP60, respectively) were obtained by different ethanol precipitations (30%, 40%, 50%, and 60%). Physicochemical properties, antioxidant activity, and emulsifying ability of HP were investigated and analyzed. Results showed that the surface structure of pectin was changed by ethanol fractional precipitation, and four fractions were low methoxy pectin. They had different monosaccharide compositions, but all rich in GalA. The Mw/Mn of CAHP30, CAHP40, CAHP50, and CAHP60 were 3.29, 2.57, 2.66, and 2.77, respectively. CAHP30 and CAHP60 had excellent emulsifying ability; moreover, CAHP60 was endowed with additional lipid antioxidant capacity and had the best thermal stability. E‐CAHP40 exhibited a property between the entangled network structure. Overall, pectin with specific properties could be obtained by different ethanol concentrations.

## Graphical abstract

Four pectin fractions were successfully obtained by different ethanol precipitation.The properties of four pectin fractions do not exhibit ethanol concentration dependence.Pectin with different characteristics can be obtained by different ethanol concentrations.

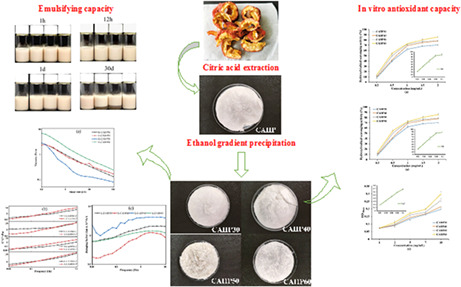

## 1 INTRODUCTION

Hawthorn (*Crataegus* spp.) is a traditional woody plant in the Rose family, and is widely distributed in Europe and North America, especially in China (Qin et al., 2022; Zhu et al., 2015). So far, there are 1000 species of the genus that have been reported. Chinese hawthorn (*Crataegus pinnatifida*), also known as mountain hawthorn, is undoubtedly the most ubiquitous species in China (Li et al., 2021; Zhu et al., 2022). Hawthorn is rich in several kinds of nutrients, such as reducing sugar, protein, vitamins, and minerals, several possible active chemical constituents have been prepared and identified from hawthorn, including flavonoids, anthocyanins, and pectin; among them, the contents of hawthorn pectin (HP) are the most abundant (Roman et al., 2021). HP which possesses unique properties, functions, and activities has aroused more research attention. Many studies indicated that HP can be widely used in food industries, pharmaceutical industries, biomedical industries, etc. (Bu et al., 2022; Chen et al., 2019; Guo et al., 2019; Li et al., 2021; Li, Fu et al., 2022; Li, Zhang et al., 2022; Qin et al., 2022; Roman et al., 2021; Sun et al., 2020; Zhang et al., 2022; Zhou et al., 2021). Moreover, the purities, physicochemical properties, chemical structure, and molecular weight depend on the extraction and purification methods of HP (Fan et al., 2022; Hui & Gao, 2022; Li et al., 2021; Zhang et al., 2021).

Generally, chromatography and ethanol gradient precipitation are the two uppermost methods used in the separation and purification of pectin (polysaccharide). Different fractions of crude pectin can be purified by different separation methods, including high‐performance liquid chromatography, gel filtration, ion exchange chromatography, etc. (Li, Yi et al., 2022). But, the chromatography purification strategies of pectin are of high cost, time consuming, and low recovery, and they are not suitable for industrial production from industrial point of view (Xu et al., 2014). Fractional precipitation with ethanol possesses the characteristics of simple, rapid, and easy concentration, and is often used in initial purification of aqueous extracts to obtain pectin (Hui & Gao, 2022). The ethanol gradient precipitation can be applied to gain polysaccharides with different molecular weights, and it is suitable for large‐scale preparation in industrial production (Hu & Goff, 2018; Naji‐Tabasi et al., 2016). Essentially, ethanol concentration is a crucial factor in the molecular weight and physicochemical properties of pectin, which may be related to the application characteristics of pectin; pectin components with different molecular weights can be obtained using this method (Chen, Liu et al., 2016; Xu et al., 2014; Zhang et al., 2018). To our knowledge, the HP was previously obtained by the ethanol precipitation method at ethanol concentration above 75%, but ethanol concentration had rarely been taken into account when preparing HP samples.

In this work, different HP fractions from hawthorn were obtained by fractional ethanol precipitation method. The physicochemical properties of pectin fractions were characterized by esterification degree, monosaccharide composition, molecular weight distribution, etc., moreover, the emulsifying properties and antioxidant activities of four HP fractions were also compared. Based on this study, pectin fractions with different characteristics could be obtained by different concentrations. The results obtained in this work will expand the application scope of HP in industrial production.

## 2 MATERIALS AND METHODS

### 2.1 Materials, pectin extraction and ethanol gradient precipitation

Dried hawthorn was obtained from Laiwu Wanbang Food Co., Ltd. All reagents used in the experiments were analytical reagents unless otherwise noted.

Fifty gram dried hawthorn was beaten with liquid at 1:30, pH was adjusted to 2.5 with the solution of 2 mol/L citric acid, and then the mixture was kept in 85°C water bath for 120 min. The extract solution was obtained by centrifuging the mixture for 15 min. The extract solution was concentrated by a rotary evaporator and then four times the volume of 95% edible alcohol was added to the concentrated solution after it was cooled to room temperature. After stirring for 10 min and depositing at 4°C for 24 h, the crude pectin was obtained by centrifugation at 5000 × *g* for 15 min.

The crude pectin was washed two to three times with 95% edible alcohol and dissolved in deionized water after removing excess alcohol. The solution was dialyzed for about 48 h, and the water was changed every 4 h. The purified pectin was finally obtained by rotary evaporation and freeze drying after the dialysis.

Five gram freeze‐dried samples were dissolved into deionized water to a concentration of 1%. Then, 95% edible alcohol was added to the sample solution to make the ethanol content in the system 30%. Thereafter, the mixture was stirred and refrigerated (about 4°C) for 24 h, then centrifuged for 15 min at 5000 × *g*, and passed through a 100 mesh sieve to obtain 30% ethanol‐precipitated pectin. The supernatants containing 40%, 50%, and 60% ethanol were prepared by adding edible alcohol to obtain ethanol‐precipitated pectin.

### 2.2 Physicochemical analysis

#### 2.2.1 Galacturonic acid, protein content and degree of esterification

The m‐hydroxybiphenyl method reported by Kintner and Van Buren (1982) was applied to analyze the content of galacturonic acid in pectin samples. The protein content in pectin was measured using the Coomassie bright blue method (Bradford, 1976). The degree of esterification was calculated by dividing the peak area at 1740 cm^−1^ by the sum of the peak areas at 1740 and 1630 cm^−1^ using FTIR spectrum (Gnanasambandam & Proctor, 2000).

#### 2.2.2 UV spectroscopic analysis

One mg/mL pectin solution was scanned using an ultraviolet spectrophotometer in the range 190–400 nm at 25°C, and UV absorbance spectrum was acquired.

#### 2.2.3 Surface morphology analysis

The Scanning Electron Microscope (SEM) (ZEISS Sigma 300) was used to gain the microstructure of pectin fractions. Dried pectin samples coated with gold were mounted onto a scanning electron microscopy specimen stub with conductive tape. Subsequently, the SEM with an accelerating voltage of 5 kV was applied to observe and photograph the characteristics of pectin fractions at ×150 magnification.

### 2.3 Molecular weight determination

The molecular weight and distribution of pectin samples were analyzed using high‐performance liquid chromatography equipped with a guard column of TSK (35 mm × 46 mm), columns of TSK gel G3000PWxl (300 mm × 4.6 mm), and TSK gel G4000PWxl (300 mm × 4.6 mm). The mobile phase was 0.1 mol/L NaNO_3_ solution containing 0.05% NaN_3_. The 3 mg/mL pectin solution was prepared using 0.05% NaN_3_ solution and was filtered by 0.45 μm water filter. The column temperature, the flow rate, the injection volume, and the elution time were 40°C, 0.4 mL/min, 20 μL, and 100 min, respectively. P‐82 pullulans standard (2 mg/mL) served as markers. The standard curve equation of P‐82 pullulan is $f\left(x\right)=-0.00036x^{2}+0.0413x^{2}-1.703x+29.37$, and *R* ^2^ = 0.9997 (Mw 1080, 6100, 9600, 21,100, 47,100, 107,000, 194,000, 337,000 Da).

### 2.4 Monosaccharide composition determination

The gas chromatographic method was used to determine the monosaccharide composition of pectin fractions (Qin et al., 2022).

### 2.5 FTIR analysis

The differences in functional groups of pectin samples were determined using the Nicolet iS10 FTIR spectrometer (Thermo Fisher Scientific; Li et al., 2018). The pectin fractions were mixed and pressed into a thin pallet with KBr, then the infrared spectrograms of samples were gained in the wavelength range 4000–400 cm^−1^.

### 2.6 Congo red

Two microliter of 2.5 mg/mL pectin sample solution was mixed evenly with 2 mL Congo red solution (80 μmol/mL), and then different volumes of NaOH solution (1 mol/mL) were slowly added into the mixture so that the concentration of NaOH in the mixture was 0.1, 0.2, 0.3, 0.4, and 0.5 mol/mL. After 10 min at 25°C, the absorption spectra in the wavelength range 190–600 nm were scanned by UV spectrophotometer and the maximum absorption wavelength was recorded.

### 2.7 Thermal analysis

Differential scanning calorimetry (DSC) was applied to analyze the thermal behavior of pectin samples (200F3, Netzsch, Germany) analysis (Sharma et al., 2015). Add 20 μL deionized water to the 5 mg pectin sample into a crucible and press the crucible lid with a crucible press, and then let it stand for about 12 h to fully wet the sample. The sample was placed in the DSC test chamber with an empty crucible as reference. Nitrogen was used to purge the crucible at a speed of 20 mL/min. The test temperature and the heating rate were 30–300°C and 10°C/min, respectively.

Thermogravimetric (TG) of pectin fractions was determined using Thermogravimetric Analyzer (DTG60A, Shimadzu, Japan). Pectin samples (5–10 mg) were placed in the sample dish and heated from 30°C to 600°C at 10°C/min. Derivative TG (DTG) curves were obtained by differentiating TG values.

### 2.8 *In vitro* antioxidant activity assays of HP

#### 2.8.1 Hydroxyl radical scavenging activity

Scavenging activity against hydroxyl radicals was tested by the method of Zhu et al. (2018) with slight modifications. One microliter pectin solution, 1 mL FeSO_4_ (0.75 mmol/L), 1 mL 0.01% H_2_O_2_, and 1 mL 0.75 mmol/L *O*‐phenanthroline–ethanol solution were mixed in order. Then, the mixture was put into water‐bath at 37°C to react. Finally, the absorbances were recorded at 536 nm. The blank control group was marked as A_p_, the H_2_O_2_ blank was marked as A_b_, the sample solution was marked as A_s_. The result was expressed as IC_50_ values. The scavenging capability was calculated by the following formula:

$$\text{Hydroxyl radical scavenging rate}\;\left(\%\right)=\frac{\mathrm{As}–\mathrm{Ap}}{\mathrm{Ab}–\mathrm{Ap}}\times 100\%$$

#### 2.8.2 Reducing power

The reducing power of samples was measured in terms of the method reported by Chen et al. (2019).

#### 2.8.3 Determination of ABTS radical scavenging rate

The ABTS radical scavenging capacity of pectin fractions was analyzed by the method of Zhang et al. (2022). The mixed solution of 7 mmol/L ABTS and 2.45 mmol/L K_2_S_2_O_8_ at 1:1 stood overnight in darkness at 25°C. And then, the mixture was diluted to the required working concentration using deionized water. Afterward, 0.3 mL pectin solution was mixed with 2 mL ABTS working solution and reacted for 10 min in the dark. The absorbance was measured at 734 nm. The test sample solution with ABTS solution, the sample solution without ABTS solution, and the ABTS solution without sample solution were marked as A_s_, A_x_, and A_0_, respectively. The scavenging rate was calculated as following:

$$\text{ABTS radical scavenging rate}\;\left(\%\right)=\left(1-\frac{\mathrm{As}–\mathrm{Ax}}{A0}\right)\times 100\%$$

### 2.9 Preparation of emulsion

Five microliter of 2% pectin solution was mixed with 5 mL corn germ oil, and the emulsion was gained by homogenization at 12,000 rpm for 2 min.

#### 2.9.1 Emulsifying properties

The method of Liu et al. (2022) was used to observe samples, and the emulsion stability during storage at 1 h, 12 h, 1 day, and 30 days after preparation was observed. The determination of emulsifying capacity (EC) and emulsion stability (ES) referred to the method of Liu et al. (2021).

#### 2.9.2 Droplet size and microscopic structure analysis

The laser particle size analyzer was applied to analyze the particle size and distribution of the emulsions. Microstructures of emulsions were obtained by an IX73 fluorescence inversion microscope system (Olympus) at 25°C.

#### 2.9.3 Rheological properties of emulsion

Rheology was measured using a modular rheometer MCR 102 (Anton Paar). For viscosity measurements, at the shear rate of 0.01–100 s^−1^ and at 25°C, stable shear was conducted on the pectin fractions. Linear viscoelastic region was tested by strain sweep experiments in the frequency range 0.01–10 Hz to obtain the G′ and G′′ of emulsions.

### 2.10 Statistical analysis

The data gained in triplicate was marked as means ± standard deviation, and processed by SPSS 26.0. Values of *p* < .05 were identified as statistically significant.

## 3 RESULTS AND DISCUSSION

### 3.1 Physicochemical property and surface morphology

**FIGURE 1:**
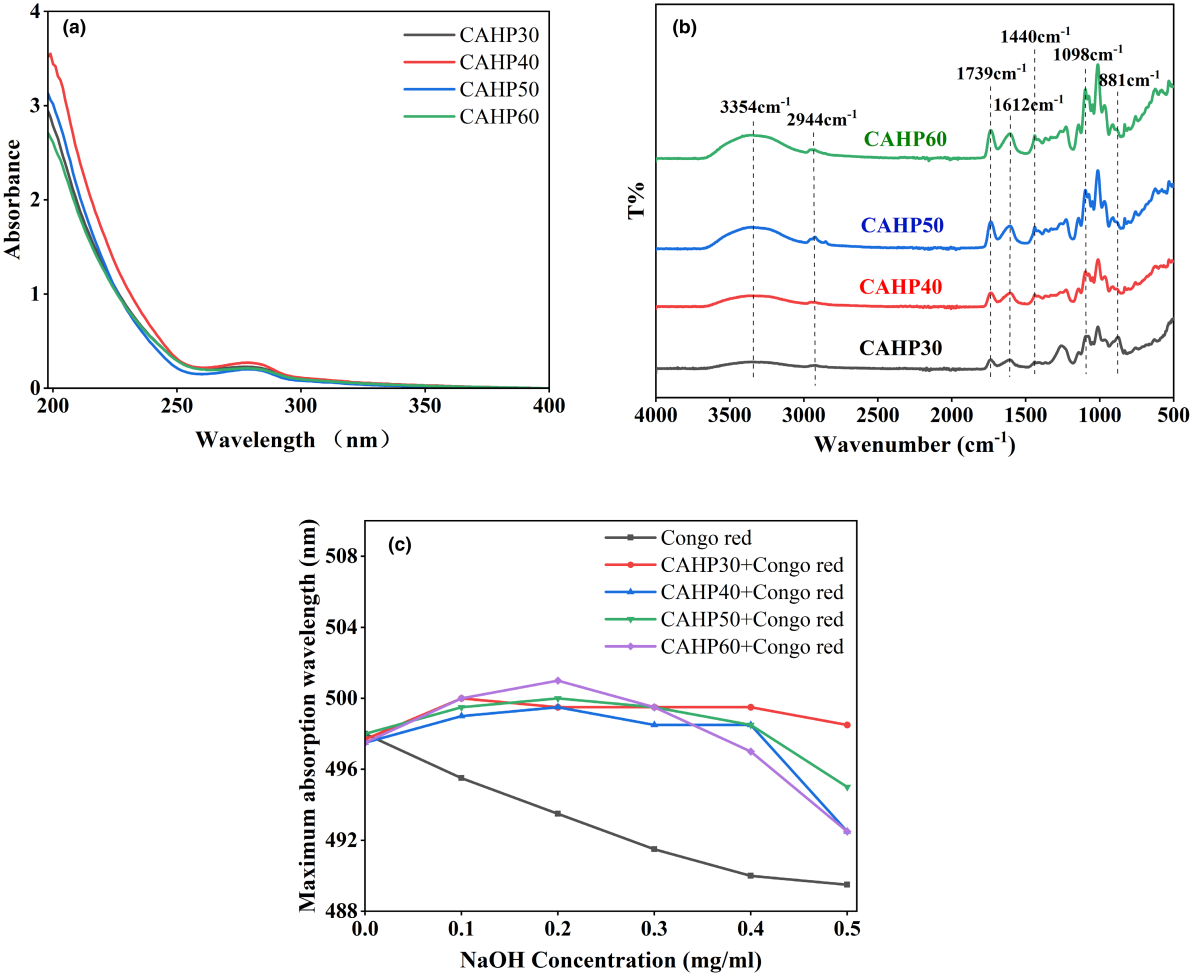
The UV spectrum of four pectin fractions (a), FTIR spectra in the range of 4000–400 cm^−1^ of four pectin fractions (b), and changes in absorption wavelength maximum of mixture of Congo red and four pectin fractions (c).

The DE value, protein content, and GalA content of four pectin fractions (CAHP30, CAHP40, CAHP50, and CAHP60) are shown in Table 1. The pectin fractions precipitated by ethanol classification were all low methoxy pectin. With the progress of ethanol classification, the DE values only changed in a small range and did not change regularly. The content of GalA in pectin samples showed a decreasing trend with the progress of ethanol gradient precipitation. After ethanol fractionation, the protein content of pectin samples was significantly decreased with the increase in ethanol concentration. The results of UV spectrum (Figure 1a) showed that pectin fractions had no absorption peak at 260 nm, which indicates that the four pectin samples did not contain nucleic acid; the absorption peak appeared at 280 nm indicating that the sample contained a small amount of protein. The above results showed that ethanol fractionation has a certain effect on the purification of pectin samples, but the protein in pectin samples cannot be completely removed (Hui & Gao, 2022).

**TABLE 1:** Basic physicochemical properties, molecular weight, and monosaccharide composition of four pectin fractions.

	CAHP30	CAHP40	CAHP50	CAHP60
DE (%)	37.69 ± 0.22^c^	37.08 ± 0.18^b^	39.80 ± 0.66^a^	37.67 ± 0.91^a^
Protein (%)	2.11 ± 0.18^b^	1.59 ± 0.36^b^	1.13 ± 0.35^bc^	0.73 ± 0.35^c^
*Molecular weight*				
Mw (kDa)	287.59 ± 1.21^a^	280.42 ± 0.62^b^	274.44 ± 0.15^b^	266 ± 2.03^b^
Mn (kDa)	87.34 ± 0.22^a^	109.26 ± 0.17^b^	103.26 ± 2.11^c^	93.13 ± 1.01^bc^
Mw/Mn	3.29 ± 0.05^b^	2.57 ± 0.12^a^	2.66 ± 0.06^a^	2.77 ± 0.08^ab^
*Monosaccharide composition*				
GalA (%)	90.27 ± 0.94^a^	89.07 ± 0.32^b^	85.17 ± 0.97^ab^	82.43 ± 0.29^c^
Rha (%)	0.51 ± 0.01^a^	0.49 ± 0.06^b^	0.53 ± 0.11^c^	0.62 ± 0.04^d^
Ara (%)	0.55 ± 0.1^b^	0.54 ± 0.08^c^	0.51 ± 0.07^a^	0.57 ± 0.01^ac^
Xyl (%)	–	0.27 ± 0.02	–	–
Man (%)	0.27 ± 0.02^c^	0.28 ± 0.01^a^	0.41 ± 0.1^b^	–
Gal (%)	0.33 ± 0.02^a^	0.35 ± 0.02^a^	0.32 ± 0.04^b^	0.35 ± 0.1^c^
Glc (%)	0.28 ± 0.01^b^	0.27 ± 0.03^a^	0.27 ± 0.02^b^	0.27 ± 0.09^ab^
HG (%)	99.33 ± 0.14^a^	99.35 ± 0.23^b^	99.26 ± 0.13^ab^	99.11 ± 0.16^c^
RG‐I (%)	0.67 ± 0.04^c^	0.65 ± 0.03^b^	0.74 ± 0.02^bc^	0.89 ± 0.06^a^
(Ara + Gal)/Rha	1.73 ± 0.12^b^	1.82 ± 0.06^b^	1.57 ± 0.11^b^	1.48 ± 0.1^a^

Values with different letters (a‐d) in the same line are significantly different (*p* < 0.05).

**FIGURE 2:**
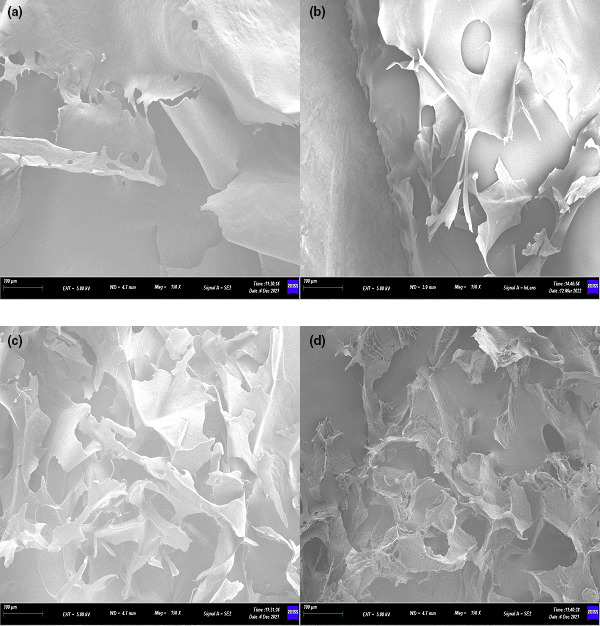
Scanning electron microphotographs (SEM) of CAHP30 (a), CAHP40 (b), CAHP50 (c), and CAHP60 (d). The magnification of (a–d) is ×150.

From Figure 2, CAHP30 had a relatively intact large‐scale structure with serrated cracks and holes on surface. CAHP40, CAHP50, and CAHP60 were incomplete pieces, and the forms have become fragmented compared to CAHP30. Among them, CAHP40 had large holes on the surface but with a relatively flat sheet structure, but all of them had been broken into different sizes. The CAHP50 and CAHP60 were no longer visible in their full mass form, becoming crumpled and curly. Therefore, we inferred that the surface morphology of pectin could be changed by ethanol fractional precipitation, and the degree of pectin surface integrity was negative with precipitation concentration (Sun et al., 2020).

### 3.2 Molecular weight and distribution of HP

Molecular weight of pectin (Table 1) decreased with the ethanol grading, indicating that HP with lower Mw can be obtained at high ethanol concentration. The results were similar to the results gained by Hui and Gao (2022). The polydispersity coefficient can be used to evaluate the molecular weight distribution of pectin samples. The larger the polydispersity coefficient, the wider the molecular weight distribution of the samples; otherwise, the narrower (Jiang et al., 2018). As shown in Table 1, the Mw/Mn of CAHP30, CAHP40, CAHP50, and CAHP60 were 3.29, 2.57, 2.66, and 2.77, respectively, indicating that CAHP30 had the widest molecular weight. The Mw/Mn of CAHP40, CAHP50, and CAHP60 ranged from 2.57 to 2.77, which proved the homogeneity of these three fractions (Xu et al., 2014).

### 3.3 Monosaccharide composition of pectin fractions

From Table 1, the content of GalA in the four fractions was above 80%, CAHP30 and CAHP50 had the same monosaccharide composition, containing rhamnose (Rha), arabinose (Ara), mannose (Man), galactose (Gal), and glucose (Glc), but the content of each monosaccharide was different. Compared with CAHP30 and CAHP50, CAHP40 contained xylose (Xyl). In addition, the branching degree and the length of pectin can be expressed by (Ara + Gal)/Rha (Qin et al., 2022). The ratio of (Ara + Gal)/Rha of CAHP30 (1.73), CAHP40 (1.82), CAHP50 (1.57), and CAHP60 (1.48) showed that the four ethanol‐precipitated pectin samples had short side chains and low degree of branching. The HG content of the four pectin samples was above 99%, indicating that all the pectin obtained by ethanol precipitation was HG type. The results were different from the HP gained previously the proportion and composition (Bu et al., 2022; Chen et al., 2019; Li, Zhang et al., 2022; Qin et al., 2022; Roman et al., 2021; Sun et al., 2020; Zhang et al., 2022), which indicated that ethanol precipitation has a certain effect on the monosaccharide conformation of pectin and CAHP30, CAHP40, CAHP50, and CAHP60 were new pectin polysaccharides.

### 3.4 Thermal analysis

**FIGURE 3:**
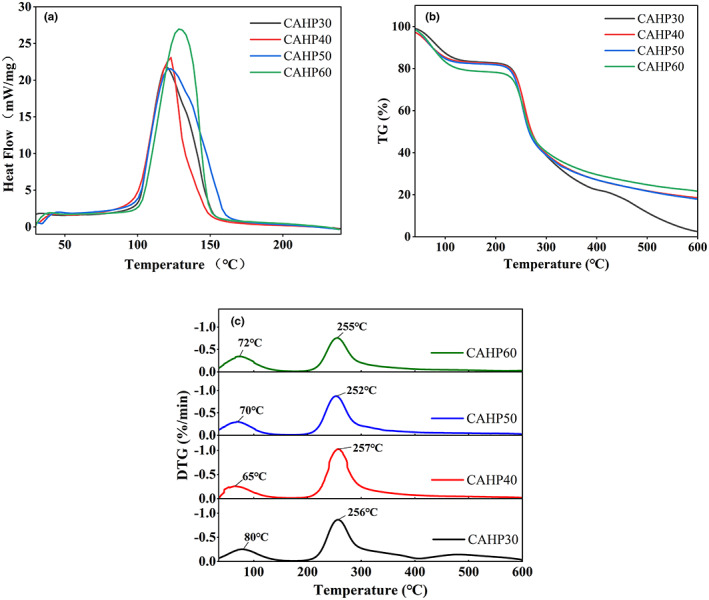
DSC thermograms (a), thermogravimetric (TG, b), and thermogravimetric (DTG, c) of four pectin fractions.

The exothermic peaks of the four pectin samples were different (Figure 3a), which reflected the unique degradation characteristics of the samples. DSC results showed that the degradation temperature of different samples (CAHP30: 119.6°C, CAHP40: 123°C, CAHP50: 122.9°C, and CAHP60: 128.3°C) and their enthalpy change (CAHP30: 3450 J/g, CAHP40: 2916 J/g, CAHP 50: 4120 J/g, and CAHP60: 4234 J/g) were different. It showed that the degradation temperature of pectin fractions increased with the increase in ethanol precipitation concentration, indicating that ethanol fractionation can improve the thermal stability of pectin, and the thermal stability was positively correlated with ethanol concentration. The reason for the change in degradation temperatures may be that the chemical composition of pectin samples was changed by ethanol fractionation, which was related to the DE value, Mw, and GalA content of the pectin samples (Sun et al., 2020). It was worth mentioning that CAHP40 had the sharpest exothermic peak, which indicated that it had the shortest melting range and concentrated molecular weight distribution. Previous data on molecular weight (Table 1) corresponded to this result. To sum up, CAHP60 was more resistant to high temperatures, which made it a potential additive for heating food.

The TG curves of the pectin samples are shown in Figure 3b. Three degradation stages were shown in the range 30°C–600°C. The first stage occurred between 40°C and 180°C, and the mass loss of pectin samples was around 18%–22%, which may be due to the evaporation of free and bound water in the sample (Qin et al., 2022). The mass loss of pectin was the largest in the second stage, which occurred between 200°C and 330°C. The depolymerization of pectin led to the degradation of the pectin galacturonic acid chain and side chain (Jiang, Qi et al., 2020; Xu et al., 2022). The third stage was between 330°C and 600°C, and the quality reduction related to the burning of aromatic carbon (Norcino et al., 2020). The mass residue at 600°C of CAHP60 was about 21.72%, the residual amount of CAHP40 and CAHP50 was about 18.58% and 17.95%, and the residual amount of CAHP30 was 2.54%, which was significantly lower than other samples.

According to the DTG curve (Figure 3c), in the first degradation stage, the peak of CAHP40 was at 65°C, which was significantly lower than that of other samples, indicating that more water was combined in CAHP40 and more mass was lost by water evaporation. The peak temperature of CAHP30 was 80°C, indicating that it had less water content. The decrease in peak temperature in the second stage was generally caused by the decrease in molecular weight due to the destruction of pectin molecules, and lower molecular weight corresponds to lower peak temperature (Jiang, Qi et al., 2020). The molecular weights of the four pectin fractions were different, but their peak temperatures varied in a very small range. We speculated that the ethanol fractional precipitation did not destroy the pectin molecules and could not cause a significant change in the peak temperature.

### 3.5 FTIR spectroscopy

Infrared spectroscopy is one of the important techniques for analyzing the primary structure of pectin. According to Figure 1b, four fractions of pectin obtained by ethanol fractional precipitation showed similar characteristic absorption peaks of polysaccharides, and the peak intensity showed a gradually increasing trend. The absorption peaks of the four pectin at 3354 cm^−1^ were caused by the tensile vibration of O–H (Li, Zhang et al., 2022). The absorption peak at 2944 cm^−1^ was the C–H stretching vibration (CH, CH_2_, and CH_3_ groups; Jiang, Xu et al., 2020). Both of these were typical of polysaccharide absorption. The absorption peaks near 1739 and 1612 cm^−1^ were the tensile vibration of esterified carbonyl C=O and the free carboxyl COO^−^. The DE values of the previous four pectin grades were calculated from these two peak areas (Muñoz‐Almagro et al., 2017). The peaks at 1440 cm^−1^ are due to the bending vibration of C–H. The smaller peaks between 900 and 1200 cm^−1^ represented the “fingerprint” region of carbohydrates (Chen et al., 2021). Weak absorption peaks at 881 cm^−1^ indicated α‐glycosidic and β‐glycosidic bonds. The results showed that ethanol grading did not change the group type of pectin (Yang et al., 2015).

### 3.6 Congo red

Pectin cannot form secondary structures like α‐helix and β‐pleated sheet, which is different from proteins. But some pectin molecules are able to form triple helix structure, which affects the biological activity of pectin. Therefore, it is necessary to figure out whether HP has a helix structure and the effect of ethanol fractionation on its triple helix structure. Congo red can be used for judging the triple helix structure of HP. Congo red can form a complex with pectin with a triple helix. In low‐concentration alkaline solution, the maximum absorption wavelength (λ_max_) of the composite shifts to a longer wavelength in the visible light range, it is called a red shift phenomenon occurs (Giese et al., 2008).

As shown in Figure 1c, compared with the Congo red solution, the pectin samples obtained by ethanol fractionation precipitation formed complexes with Congo red, and their λ_max_ increased. The λ_max_ of CAHP30 reached the maximum at the concentration of 0.1 mol/L of NaOH, and that of CAHP40, CAHP50, and CAHP60 reached the maximum at the concentration of 0.2 mol/L of NaOH. Then, with the increase in NaOH concentration, the *λ*_max_ of the four fractions decreased, but it was always higher than the blank group. The results showed that the four different fractions had triple helix structure. Further analysis of Figure 1c showed that among the four samples, the wavelength of the red shift of CAHP60 was 7.5 nm, which was greater than CAHP30 (4.5 nm), CAHP40 (6 nm), and CAHP50 (6.5 nm). Results showed that ethanol fractionation had an effect on the molecular conformation of pectin samples, mainly because the conformation of the pectin molecule was related to its molecular weight distribution (Wang & Li, 2019), and ethanol fractionation affected the molecular weight distribution of HP.

### 3.7 Emulsifying properties of pectin samples

#### 3.7.1 Appearance, microscopy, and particle size analysis of emulsion

**FIGURE 4:**
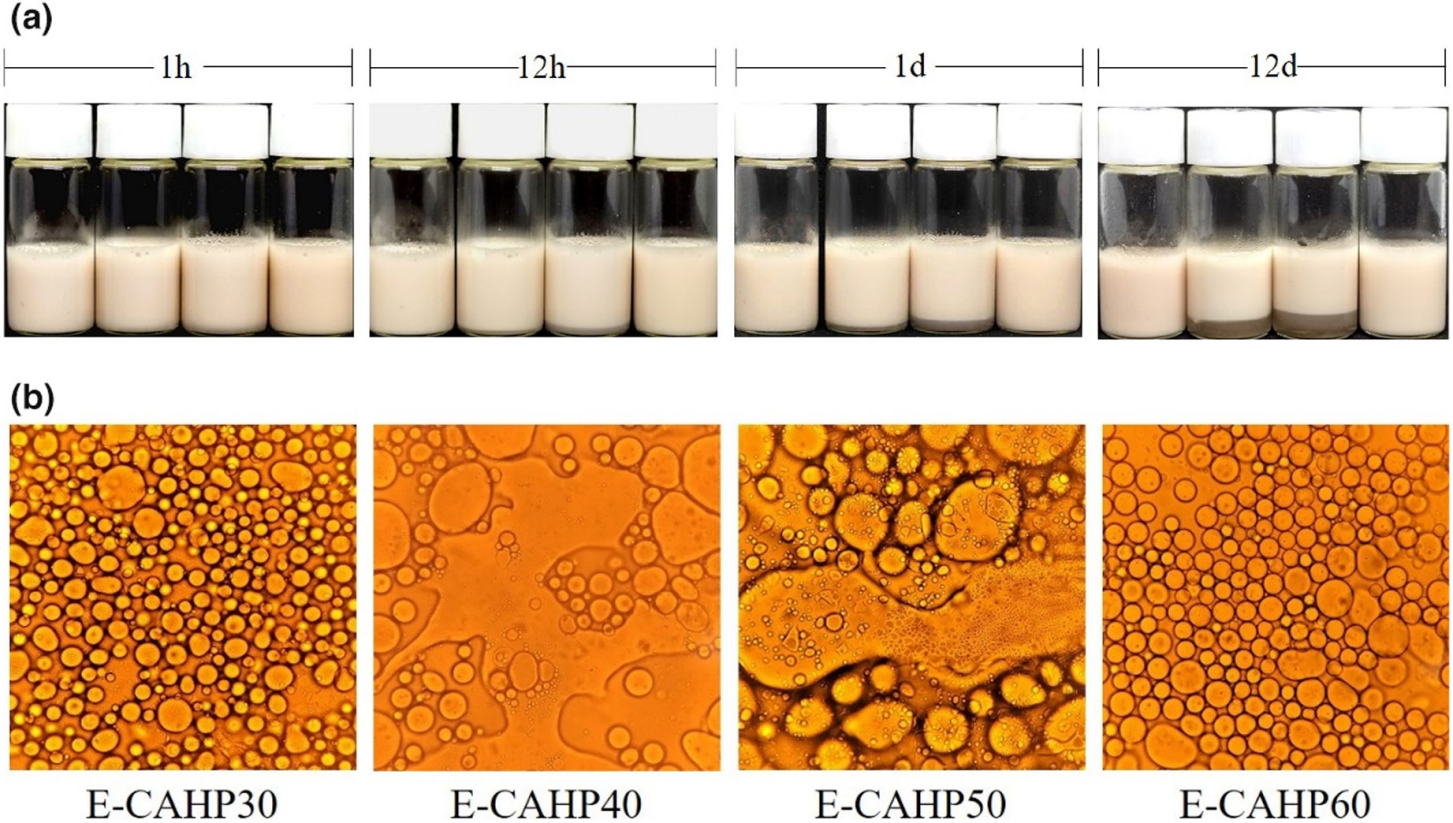
Digital photos during storage with CAHP30, CAHP40, CAHP50, and CAHP60 stabilized oil‐in‐water emulsions (a) and optical micrographs preserved for 30 days (b).

As shown in Figure 4a, no demulsification occurred during the preparation of all emulsions. E‐CAHP40 and E‐CAHP50 showed a tendency of phase separation after 1 h of preparation. After the storage of 12 h, the phase separation of E‐CAHP40 and E‐CAHP50 was slight. The degree of phase separation of E‐CAHP40 and E‐CAHP50 showed an increasing trend after 1 day of storage, while E‐CAHP30 and E‐CAHP60 had no significant change. After storage for 30 days, E‐CAHP40 and E‐CAHP50 had obvious continuous‐phase precipitation, and E‐CAHP30 and E‐CAHP60 remained stable throughout the observation period. The results showed that CAHP30 and CAHP60 had excellent emulsifying ability, while CAHP40 and CAHP50 were not suitable for use as emulsifiers.

**FIGURE 5:**
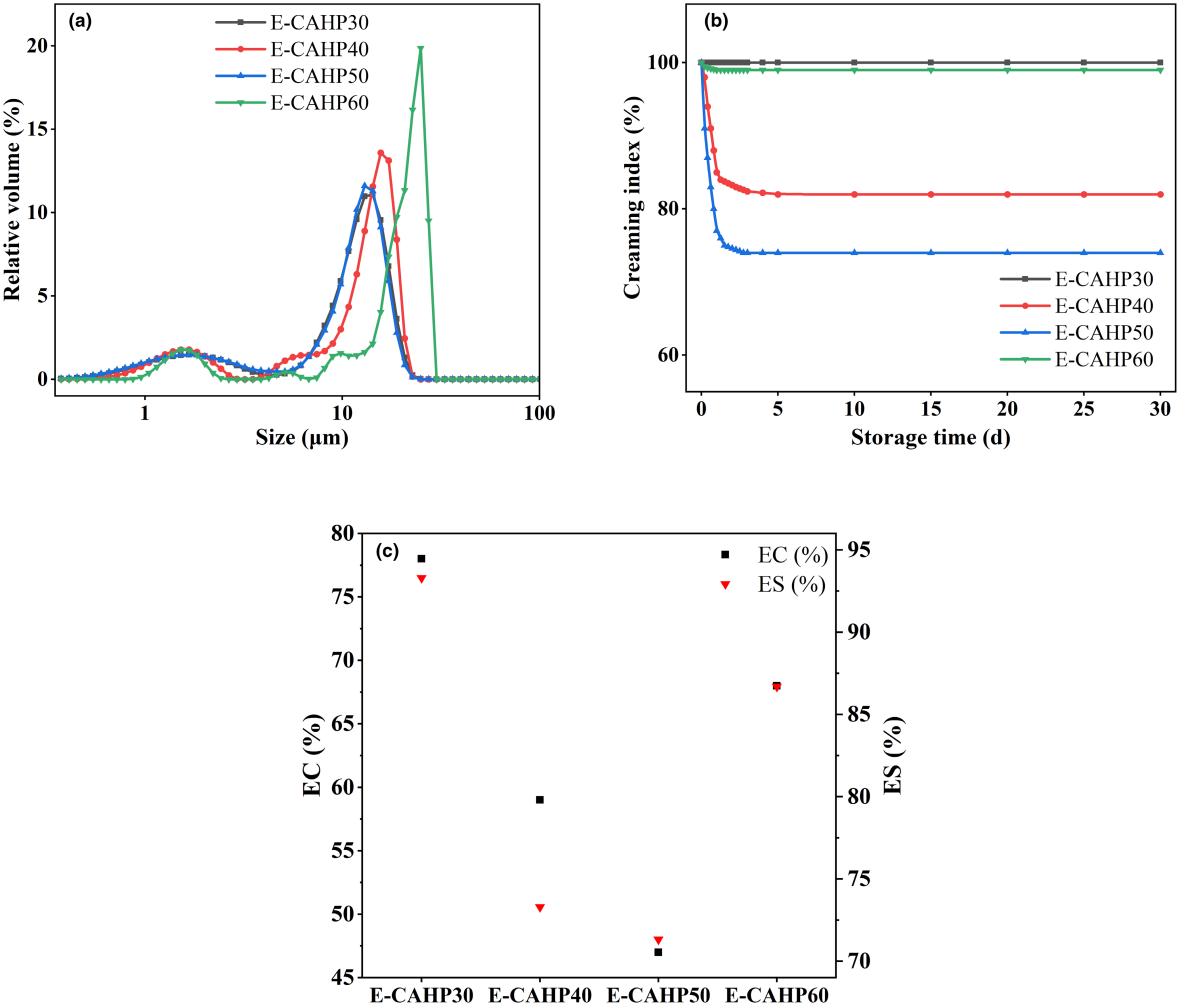
The particle size distribution of emulsion after storage for 30 days (a), creaming index (CI) of emulsion prepared with different pectin fractions (b), and emulsifying capacity (EC) and emulsifying stability (ES) of emulsion prepared with different pectin fractions (c).

Microscopic images of the emulsions after storage for 30 days are shown in Figure 4b. We found that E‐CAHP40 and E‐CAHP50 had a demulsification phenomenon, which indicated that CAHP40 and CAHP50 had poor emulsifying properties. E‐CAHP30 had smaller droplet size than E‐CAHP60, and the study demonstrated that the smaller droplet size is beneficial to the stability of the emulsion (Jia et al., 2015). The particle size of all sample emulsions after storage for 30 days was less than 50 μm, and the droplet size distribution was generally consistent with the stability and microstructure of the emulsion (Figure 5a).

#### 3.7.2 Creaming index, emulsifying capacity, and emulsifying stability of pectin

Figure 5b showed the changing trend of the creaming index of the emulsion within 30 days of storage. The CI values showed a rapid decline trend within 24 h after the preparation of the emulsion, after which the decline rate tended to be gentle until it remained stable. All emulsions were stable after 1–5 days of storage until the end of the observation period. The CI value of E‐CAHP30 always maintained a stable state, and the CI of E‐CAHP60 had a downward trend. Compared with them, the CI values of E‐CAHP40 and E‐CAHP50 decreased significantly (*p* < 0.05), and the CI decline rate and decline range of E‐CAHP50 were higher than E‐CAHP40. It was found that the viscosity, water‐holding capacity, and steric structure of the emulsifier influenced the rate of decline and the final value of CI (Sun et al., 2019). Among them, the viscosity of the emulsifier had a more significant influence on CI. The higher the viscosity, the higher the CI, and the more stable the emulsion. The reason was that more emulsifiers can reduce the tension of the water/oil interface and stabilize the droplets, and it also attenuated the movement of the continuous phase (Cai et al., 2019). In comparison of emulsifying capacity (EC) and emulsifying stability (ES) of four samples (Figure 5c), both CAHP30 and CAHP60 had ideal emulsifying properties, while CAHP40 and CAHP50 had low EC and ES levels and were not suitable for use as emulsifiers.

The results showed that CAHP30 and CAHP60 had outstanding emulsifying abilities. The emulsifying ability of pectin is related to RG‐I and galacturonic acid domain, protein content, and neutral sugar side chain of acetyl group (Zhang et al., 2020). Roman et al. (2021) found that the EC of purified citrus and HP was positively correlated with the proportion of HG and negatively correlated with the proportion of RG‐I. Schmidt et al. (2015) reported that protein bonding can enhance the interfacial adsorption of macromolecules at the oil–water interface, thus making the emulsion more stable, so the protein content may have a certain relationship with ES. However, the experimental results of Roman et al. (2021) showed that CP1 and MI + E had almost the same protein content, but their ES had certain differences. Higher protein content, HG ratio, and lower DE value can significantly improve the emulsifying properties of HP.

### 3.8 Rheological properties of emulsion

**FIGURE 6:**
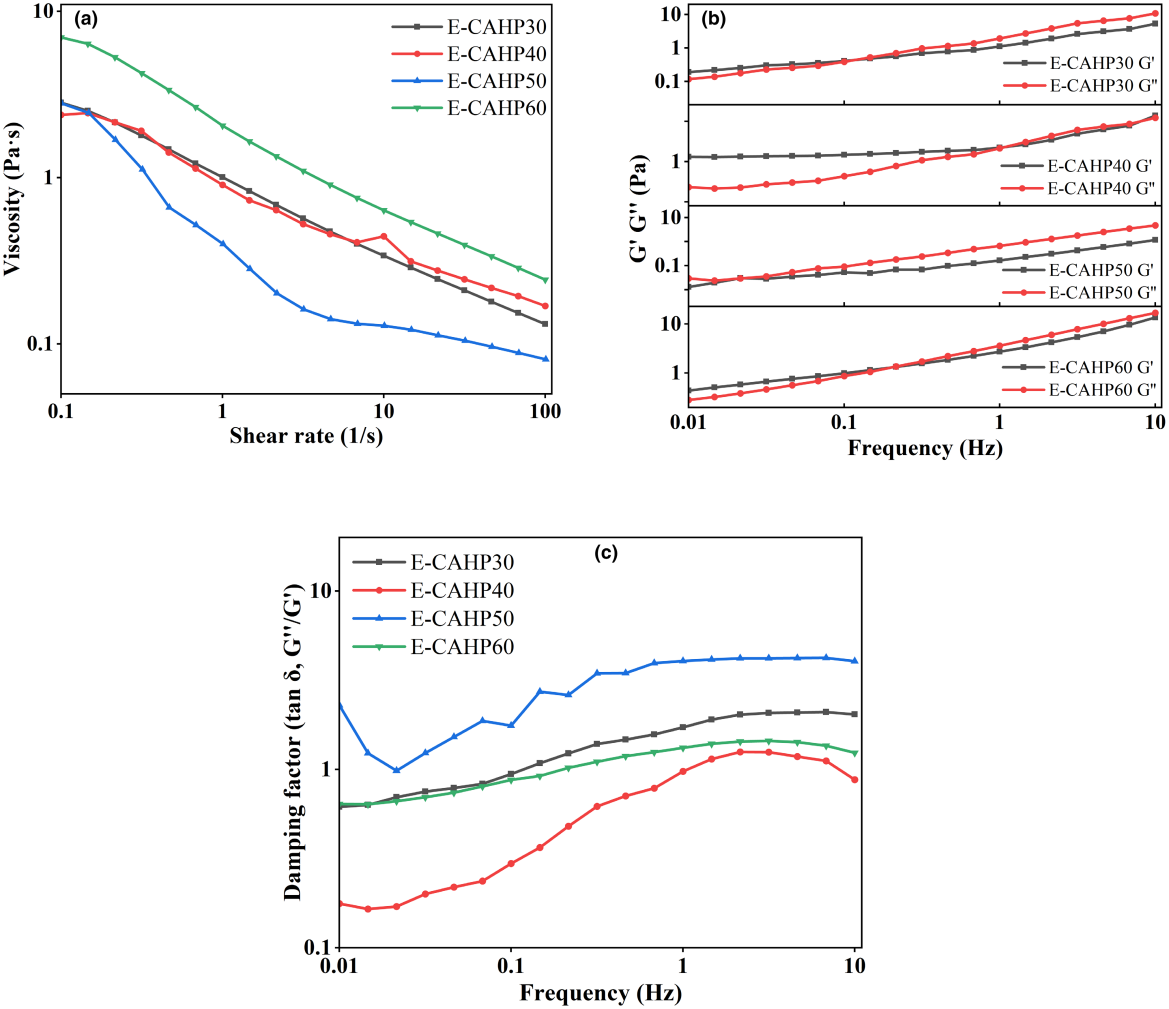
Viscosity of emulsion prepared with four pectin fractions (a), the frequency dependence of storage modulus and loss modulus (b), and damping factor (c) of emulsion prepared with four fractions.

As shown in Figure 6a, the emulsions prepared from different pectin samples have different viscosities, and the viscosity curves of the four sample emulsions all show shear‐thinning characteristics. But for E‐CAHP40, with increasing shear rate, we observed three stages, the shear thinning‐shear thickening‐shear thinning curve (Jia et al., 2015). Ren et al. (2020) demonstrated that high‐viscosity continuous phase can inhibit droplet motion and phase separation, resulting in better stability of the resulting emulsion. Increasing the concentration of protein in the aqueous phase or the rearrangement of droplets caused by excessive shear forces can enhance the intermolecular interactions, resulting in a corresponding increase in the apparent viscosity of the emulsion (Jiang, Xu et al., 2020; Liu et al., 2019). Pectin with high molecular weight had a longer molecular chain than low‐molecular‐weight pectin, it had more active binding sites, and it can form an osmotic network structure with high viscosity and elastic modulus faster (Cao et al., 2020).

According to Figure 6b, it can be seen that with the change in ethanol concentration, G′ and G′′ of E‐CAHP30, E‐CAHP50, and E‐CAHP60 all intersected at lower frequency. Below this frequency, G′ was higher than G′′, and above this frequency, G′′ was greater than G′, indicating that the emulsion was transformed from elastomer to solution. The G′ of E‐CAHP40 was higher than that of G′′, but there were intersection points between G′ and G′′ at high frequency, which indicated that E‐CAHP40 exhibited a property between the entangled network structure and the weak gel structure, and the weak gel structure was easily destroyed into the entangled network structure at high frequency. The loss tangent angle (tan δ) was the ratio of G′ to G′′. When tan δ < 1, the system mainly exhibited elastic characteristics, and when tan δ > 1, the system mainly exhibited viscous characteristics. Figure 6c showed that E‐CAHP50 almost presented solution characteristics in the process of increasing frequency, E‐CAHP40 showed that weak gel structure was destroyed, and E‐CAHP30 and E‐CAHP60 showed a relatively stable trend of transition from elastomer to solution.

### 3.9 *In vitro* antioxidant activity assays of HP

**FIGURE 7:**
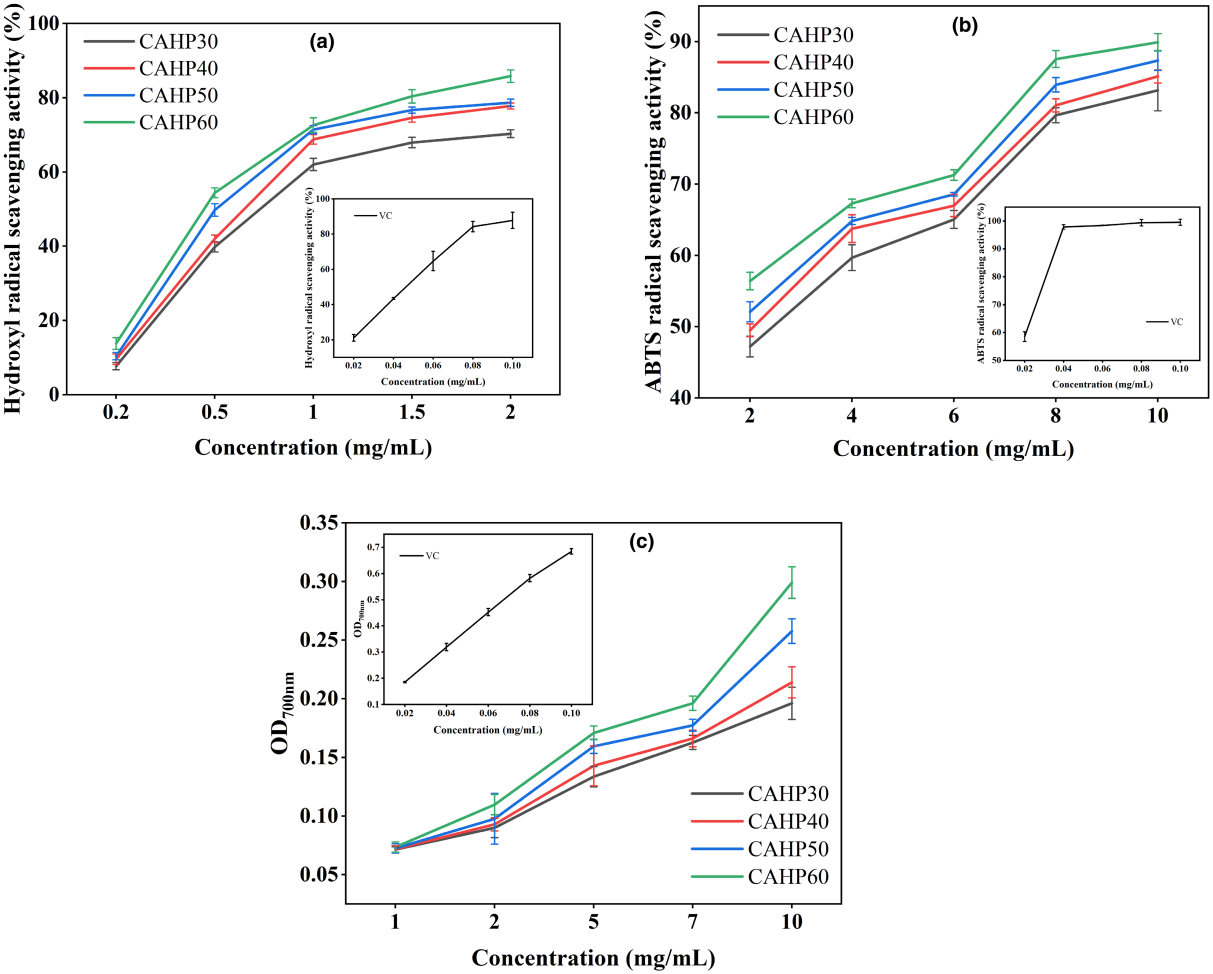
Effect of ethanol fractionation on hydroxyl radical scavenging capacity (a), ABTS radical scavenging capacity (b), and reducing power (c) of pectin fractions.

As shown in Figure 7a, the hydroxyl radical scavenging rate of all pectin fractions showed an escalating trend with the rise of concentration of pectin samples from 0.2 to 2 mg/mL. The IC_50_ values of pectin samples were 0.041 mg/mL (VC), 0.831 mg/mL (CAHP30), 0.697 mg/mL (CAHP40), 0.623 mg/mL (CAHP50), and 0.551 mg/mL (CAHP60), indicating that the hydroxyl radical scavenging rate was improved with the ethanol grading. Research by Li et al. (2013) and Wang et al. (2022), respectively, demonstrated that polysaccharides with low molecular and high GalA content had better hydroxyl radical scavenging ability. Polysaccharides with low molecular weight cause more hydroxyl groups to be exposed in pectin, and the higher content of GalA made the electrophilic group of pectin more abundant, which was beneficial to the release of hydrogen in O–H bonds (Wang et al., 2022; Yan et al., 2016).

The ABTS radical clearance rate of pectin presented concentration dependence (Figure 7b). The IC_50_ values of the samples were 0.016 mg/mL (VC), 2.547 mg/mL (CAHP30), 1.944 mg/mL (CAHP40), 1.618 mg/mL (CAHP50), and 1.188 mg/mL (CAHP60), which indicated that ethanol fractionation could significantly promote the ABTS clearance rate, and the ABTS scavenging rate was positively correlated with ethanol concentration. The ABTS scavenging rate of pectin was correlated with the molecular weight, mainly because the lower molecular weight exposes some of the active groups of pectin, producing more hydrogen that helps to scour free radicals (Wu et al., 2018).

The absorbance of pectin was positively correlated with its reducing power, and higher absorbance indicated strong reducing power and antioxidant power (Chen & Huang, 2019). As shown in Figure 7c, the absorbance value of samples reached the maximum at 10 mg/mL and absorbance value of CAHP60 was 0.299, the results showed that CAHP60 had better antioxidant capacity, indicating that the reduction of molecular weight of pectin promoted the improvement of its reducing power (Chen, Shi et al., 2016), the reason is that the reduction in molecular weight of pectin causes structural changes that expose more reducing ends and produce more reductones, which exhibit antioxidant activity by providing hydrogen atoms (Chen, Shi et al., 2016; Li et al., 2013; Sun et al., 2020).

## 4 CONCLUSION

In this study, HP was extracted by citric acid extraction, four pectin fractions (CAHP30, CAHP40, CAHP50, and CAHP60) were obtained by ethanol fractional precipitation, and the emulsifying and antioxidant capacity were analyzed. The results showed that the properties of the components obtained by ethanol fractional precipitation did not exhibit concentration dependence. Results proved that four fractions were low methoxy pectin and were rich in GalA, but differed in monosaccharide composition. In particular, E‐CAHP30 and E‐CAHP60 had excellent EC and ES. It is found that higher HG ratio and protein content, and lower RG‐I ratio and DE value had positive effects on the emulsion stability of pectin, and CAHP30 showed better emulsifying properties overall. It is worth mentioning that the excellent antioxidant capacity of CAHP60 can effectively protect the emulsion and resist lipid oxidation. These results showed that HP gained via different ethanol concentrations can be taken as new polysaccharide emulsifiers and delivery materials, but the practical application in industrial production still needs further research.

## CONFLICT INTEREST STATEMENT

The authors declare no conflict of interest related to the publication of this manuscript.

## ETHICS STATEMENT

This study does not involve human or animal experiments.

## CONSENT FOR PUBLICATION

All the authors of this manuscript gave their consent to publish.

## Data Availability

The data that support the findings of this study are available from the corresponding author upon reasonable request.
